# Structural Characteristics and Antioxidative Capability of the Soluble Polysaccharides Present in *Dictyophora indusiata* (Vent. Ex Pers.) Fish Phallaceae

**DOI:** 10.1093/ecam/neq041

**Published:** 2011-06-15

**Authors:** Yaw-Bee Ker, Kuan-Chou Chen, Chiung-Chi Peng, Chiu-Lan Hsieh, Robert Y. Peng

**Affiliations:** ^1^Department of Food and Nutrition, Hungkuang University, Taichung Hsien, Taiwan; ^2^Department of Urology, Taipei Medical University Shuang Ho Hospital, Taipei, Taiwan; ^3^Graduate Institute of Rehabilitation Science, Taiwan; ^4^School of Physical Therapy, College of Health Care, China Medical University, Taichung, Taiwan; ^5^Graduate Institute of Biotechnology, National Chang-Hua University of Education, 1 jin-De Road, Changhua 500, Taiwan; ^6^Research Institute of Biotechnology, Hungkuang University, 34 Chung-Chie Road, Shalu Country, Taichung Hsien 43302, Taiwan; ^7^Research Institute of Medical Sciences, Taipei Medical University, Taipei, Taiwan

## Abstract

*Dictyophora indusiata* (Vent. ex Pers.) Fish Phallaceae (Chinese name Zhu-Sūn, the bamboo fungi) has been used as a medicinal mushroom to treat many inflammatory, gastric and neural diseases since 618 AD in China. We hypothesize that the soluble polysaccharides (SP) present in *D. indusiata* and their monosaccharide profiles can act as an important role affecting the antioxidative capability, which in turn would influence the biological activity involving anti-inflammatory, immune enhancing and anticancer. We obtained six SP fractions and designated them as D1, a galactoglucan; D2, a galactan; D3, the isoelectrically precipitated riboglucan from 2% NaOH; D4, a myoinositol; D5 and D6, the mannogalactans. The total SP accounted for 37.44% w/w, their molecular weight (MW) ranged within 801–4656 kDa. D3, having the smallest MW 801 kDa, exhibited the most potent scavenging effect against the *α*,*α*-diphenyl-*β*-picrylhydrazyl, •OH^−^, and •O_2_^−^ radicals, yielding IC_50_ values 0.11, 1.02 and 0.64 mg mL^−1^, respectively. Thus we have confirmed our hypothesis that the bioactivity of *D. indusiata* is related in majority, if not entirely, to its soluble polysaccharide type regarding the MW and monosaccharide profiles.

## 1. Introduction

**Figure 1:**
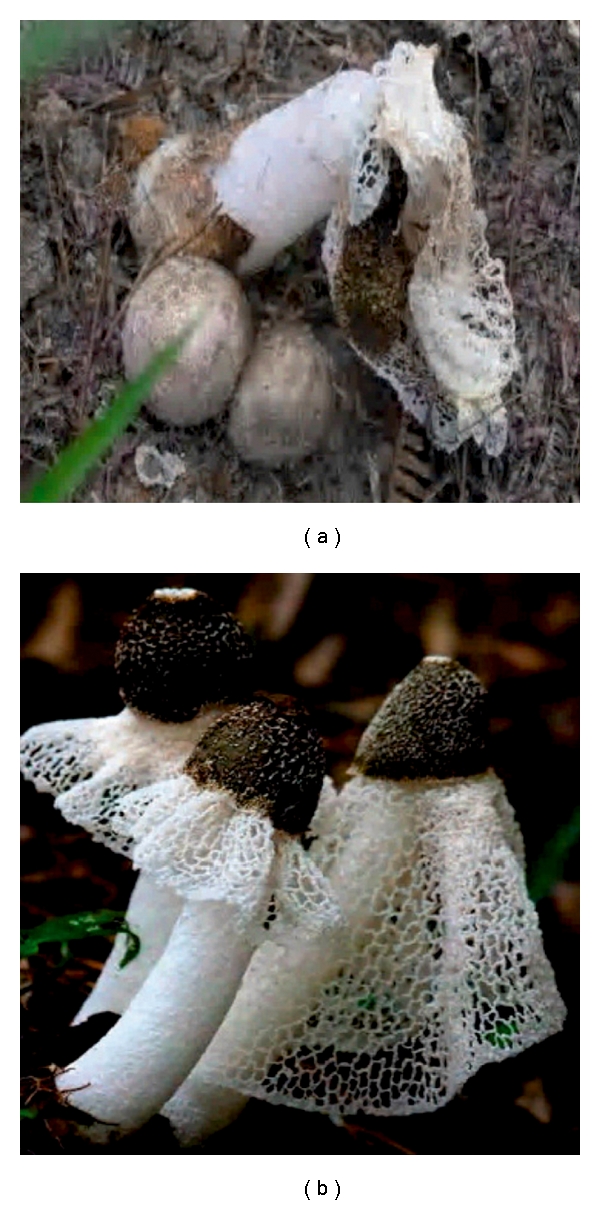
Fruiting bodies (a) and fungal mycelia (b) of *D. indusiata* (Vent. Ex Pers.) Fish Phallaceae.

*Dictyophora indusiata* (Vent. Ex Pers.) Fish Phallaceae (Chinese name Zhu Sūn, meaning the bamboo mushroom), synonymously called *Phallus indusiatus*, is frequently used under the name Veiled lady mushroom (Figure 1). The folkloric consumption of *D. indusiata* in the ancient China began around 618 AD, which pointed mainly to the nutritional bioactivities, like benefits to eyes and tonics to cardiovascular systems; and partially to the medicinal effect like mental tranquilization, antitumor, and tonics, and so forth, [1–4]. It consists of seven essential amino acids, 12 precious metallic ions and rather high content of vitamin E [5, 6], *β*-carotene, thiamine, riboflavin, nicotinic acid, l-ascorbic acid, calcium and phosphate [7, 8].

A review by Lindequist indicted that many edible mushrooms exhibit a broad spectrum of bioactivities that are beneficially acting as a complementary alternative medicine (CAM) [9]. As often cited, the therapeutic efficacy of mushrooms includes antibacterial, antifungal, anti-multiresistant bacterial, antiviral, immunomodulating, antitumor or antitumor adjuvant, cytostatic, immunosuppressive and anti-allergic, antioxidative and anti-inflammatory, antiatherosclerogenic, hypoglycemic, hepatoprotective, and neurotrophic functions [9].

The mannan (molecular weight (MW) 620 kDa) present in *D. indusiata* exhibited potent anticancer bioactivity [10]. Hara et al. isolated two soluble glucans, characteristically having *β*-1→6 branches linked to *β*-(1→3)-d-glucosan main frames [11, 12] and exhibiting potent anti-inflammatory [11, 13] as well as anticancer activity [14]. Conversely, Namba et al. and Kodama et al. successively found in *Grifola frondosa* (maitake) the antitumor and NK cells activating polysaccharides that possessed 1→6 main frame of glucan with 1→3 branching side chains (Namba et al., 1987; Kodama et al., 2003) [15, 16].

Later, Hara et al. identified a new glucan (MW 510 kDa) [17] and a new (1→3)-*α*-d-mannan [18]. Lin isolated a polysaccharide (Di-S2P) with a homologous MW 870 kDa consisting of monosaccharide d-glucose : d-galactose : d-mannose : xylose in a molar ratio 1.62 : 1.87 : 100 : 0.93 [19]. More recently, Wang et al. isolated a water-soluble triple helical glucan (PD3) having MW 510 kDa [20]. These polysaccharides effectively induced cell mitosis by upregulating colony forming stimulating factor [21]. Wei indicated that *D. indusiata* is an alkaline food in nature, which neutralizes the acidosis in the mid-aged and the senescent [22], good for the hypertensive, hypercholesterolemia and hyperlipidemia. It reduced levels of serum TG, TC and LDL-C, and raised HDL-C [23]. The Miaw people (a clan of China) use it to treat injuries and pains, cough, dysentery, enteritis, leukemia and the feeble [22]. The polysaccharides contained in *D. indusiata* has revealed to be effective as anti-tumor, anti-agglutinating, anti-inflammatory, immune-enhancer, and anti-hyperglycemic. Furthermore, the short skirt Zhu Sun is a good superoxide anion scavenger; it protects the cellular membrane lipoproteins from peroxidation, a mechanism interpreting the anti-cancer and immune-enhancing effect [22]. Clinically, *D. indusiata* is often prescribed in Chinese clinical medicine to treat laryngitis, leucorrhea, fever and oligourea, diarrhea, hypertension, cough, and hyperlipidemia, and more recently in the complimentary anticancer therapy [13, 21]. Extract of *D. indusia* had showed strong antimicrobial effects on bacteria but weak on microzymes and molds [24]. The radiation protective effect of *D. indusiata* was studied by Guo et al. [25]. The thymus and pancreatic index, CD4^+^, CD16, CD57 and interleukin 2 were all improved, while CD8^+^ was decreased [25]. Ke and Lin obtained a glycoprotein DIGP-2, having the molar ratio d-galactose : d-glucose : d-mannose = 0.78 : 2.12 : 1.00, which was shown to inhibit 36.2% of Sarcoma 180 cell-line viability [26]. In addition, Kawagishi et al. and Lee et al., separately identified a total of five neurogrowth factors [27, 28]. Ishiyama et al. discovered five monoterpene alcohols [29]. Sharma et al. discovered the anti-tyrosinase component 5-(hydroxymethyl)-2-furfural (HMF), a noncompetitive inhibitor for the oxidation of l-3,4-dihydroxyphenylalanine (l-DOPA) [30]. Mau et al. studied its antioxidative capability [31]. Moreover, the extract of *Dictyophora* was shown to be effective antimutagenics [32].

**Figure 2:**
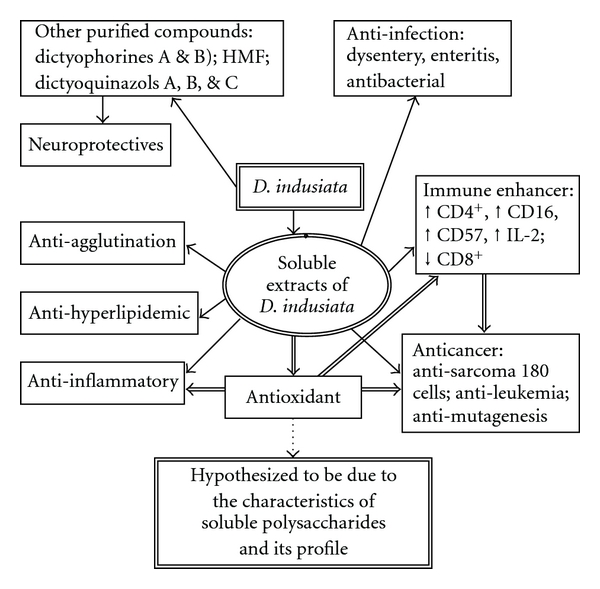
The scheme showing the hypothesis to be confirmed by the experiment. The extract of *D. indusiata* exhibits potent hypolipidemic (i), anti-bacterial (ii), anti-agglutination (iii), immune-ehancing (iv), anticancer (v), anti-inflammatory (vi) and antioxidative (vii) bioactivities. We hypothesize that the bioactivities (iv)–(vi) are relevantly affected by the antioxidative capability, which in turn would be closely related with the soluble polysaccharides present in *D. indusiata* and its monosaccharide profiles.

As is well known, the antioxidative capability may be relevantly related with its immune-enhacing, anti-inflammatory and anticancer bioactivities. We hypothesize that the SP present in *D. indusiata* and their monosaccharide profiles can act as an important role affecting the antioxidative capability, which in turn would influence the biological activity as mentioned (Figure 2).

## 2. Methods

### 2.1. Chemicals

H_2_O_2_ (1 mM) and KO_2_ (as source of 1 mM •O_2_^−^) were products of the Sigma-Aldrich Chemical Co. (Milwaukee, WI, USA). Standard dextrans and DPPH (*α*,*α*-diphenyl-*β*-picrylhydrazyl) radical methanolic DMSO solution (0.5 mM) were manufactured by the Sigma Aldrich Co. (St. Louis, MO, USA). 2-[6-(4′-Amino) phenoxy-3H-xanthen-3-on-9-yl] benzoic acid (APF) was provided by the Daiichi Pure Chemicals (Tokyo, Japan).

### 2.2. Protein Bioassay Kit

BCA protein assay kit (#23227) was a product of Pierce Rockford Co. (IL, USA).

### 2.3. Source of *D. Indusiata* (Vent. ex Pers.) Fish Phallaceae

Desiccated fruiting bodies of *D. indusiata* (Vent. ex Pers.) Fish were purchased directly from the local supplier at Ningdeh Hsien of Fujian Province of China. A voucher was attached as a supplement to the journal editorship.

### 2.4. Proximate Compositional Analysis

The proximate analysis was performed according to the methods described in AOAC [33].

### 2.5. Fractionation of Soluble Polysaccharides

**Figure 3:**
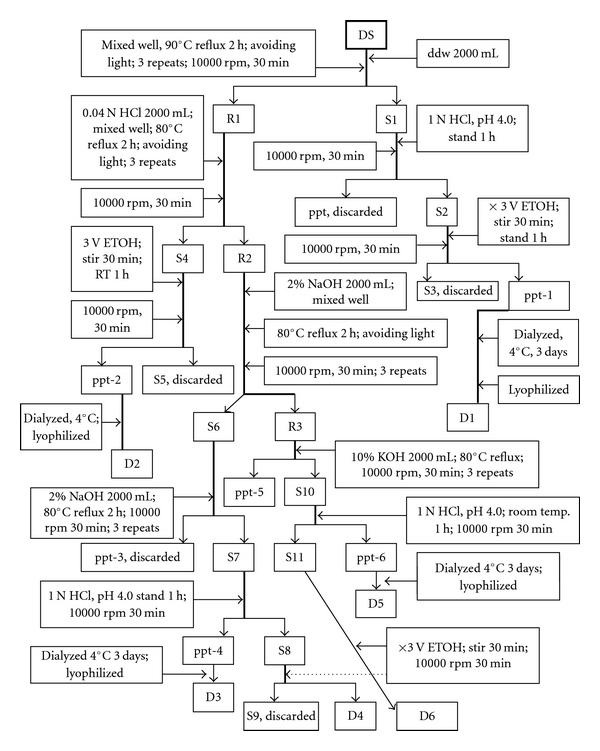
Flow chart showing the extraction protocol for isolation of soluble polysaccharides from the fruiting bodies of *D. indusiata* (Vent. Ex Pers.) Fish Phallaceae. DS: sample of desiccated *D. indusiata* fruiting body.

The fractionation of different SP from the fruiting bodies was performed according to the flowchart shown in Figure 3 [34].

#### 2.5.1. The Soluble Polysaccharide Fraction D1

Briefly, desiccated fruiting bodies of *D. indusiata* (100 g) (DS) was extracted three times while avoiding direct sunlight by refluxing with 2000 mL of double distilled water at 90°C with constant stirring at 400 × g for 2 h. On cooling, the solution was centrifuged at 10 000 × g for 30 min. The supernatant (S1) was collected. The extraction was repeated three times. The residue (R1) was kept for further treatment. The supernatants were combined and acidified with 1 N HCl to pH 4. In case that the precipitation should occur instantaneously, centrifuge the solution immediately at 10 000 × g to remove the first precipitate. Otherwise, to the solution (S2) a 3-fold volume of ethanol was added, stirred for 30 min, left to stand at ambient temperature for 1 h and centrifuged at 10 000 × g for 30 min. The precipitate (ppt-1) occurred in solvent ethanol/hot water (3 : 1) was collected, dialyzed at 4°C for 3 days, lyophilized and weighed (SP D1) (Figure 2).

#### 2.5.2. The Soluble Polysaccharide Fraction D2

To the residue (R1) 2000 mL of 0.04 N HCl were added and mixed well. The mixture was refluxed while avoiding direct light sunlight at 80°C for 2 h. The extraction was repeated three times, combined, and centrifuged at 10 000 × g for 30 min to collect the precipitate (R2). To the supernatant (S4) a 3-fold volume of ethanol was added, stirred at room temperature, left to stand for 1 h and centrifuged at 10 000 × g for 30 min to collect the precipitate (ppt-2). The supernatant (S5) was discarded. The ppt-2 obtained in the mixed solvent ethanol/0.04N HCl (3 : 1) was dialyzed at 4°C, lyophilized and weighed to obtain the SP D2 (Figure 2).

#### 2.5.3. The Soluble Polysaccharide Fraction D5

To the residue R2 2000 mL of 2% NaOH were added and mixed well. The mixture was refluxed while avoiding direct sunlight at 80°C for 2 h. The extraction was repeated for three times. The extracts were combined, cooled, and subjected to centrifugation at 10 000 × g for 30 min. The precipitate (ppt-5) was discarded (Figure 2). To the supernatant (S10) 1 N HCl was added to adjust the pH to 4.0. The acidified solution was left to stand at ambient temperature for 1 h. The mixture was centrifuged at 10 000 × g to collect the isoelectric precipitate from 10% KOH (ppt-6), which was dialyzed, lyophilized and weighed to obtain the SP D5 (Figure 2).

#### 2.5.4. The Soluble Polysaccharide Fraction D6

To the supernatant (S11) 3-fold volume of ethanol was added. The solution was stirred at ambient temperature for 30 min and centrifuged at 10 000 × g. The precipitate obtained in the ethanol/10% KOH (3 : 1) mixed solvent was collected, lyophilized and weighed to obtain the SP D6 (Figure 2).

#### 2.5.5. The Soluble Polysaccharide Fraction D3

Alternatively, to the supernatant S6 2000 mL of NaOH was added and refluxed at 80°C for 2 h. The mixture was left to stand at ambient temperature for 1 h and centrifuged at 10 000 × g to obtain the supernatant (S7). The precipitate (ppt-3) was discarded (Figure 2). An amount of 1 N HCl was used to adjust the pH value of S7 to 4.0. The acidified solution was left to stand at ambient temperature for 1 h and centrifuged at 10 000 × g for 30 min. The precipitate occurred by the isoelectric precipitation from 2% NaOH (ppt-4) was collected, dialyzed, lyophilized and weighed to obtain the SP D3 (Figure 2).

#### 2.5.6. The Soluble Polysaccharide Fraction D4

To the supernatant (S8) a 3-fold volume of ethanol was added and stirred for 30 min. The solution was centrifuged at 10 000 × g for 30 min to obtain the precipitate. The supernatant (S9) was discarded (Figure 2). The precipitate collected from the ethanol/2% NaOH (3 : 1) was dialyzed, lyophilized and weighed to obtain the SP D4 (Figure 2).

### 2.6. Determination of the Carbohydrate Content

The carbohydrate content in each fraction was determined by the phenol-H_2_SO_4_ method [35].

### 2.7. Determination of the Protein Content

The protein content in each fraction was determined by the bicinchoninic acid (BCA) protein assay kit (#23227, Pierce Rockford, IL, USA) following the manufacturer's instructions.

### 2.8. GPC Sieving

To 20 mg of each fraction (D1–D6) 1 mL of 10% NaOH and sufficient double distilled water were added to make a final volume of 5 mL. The mixture was thoroughly agitated to facilitate the dissolution and centrifuged at 2500 × g for 10 min to remove the insoluble precipitate. The supernatant was decanted, each 3 mL of which was eluted with 0.05 N NaOH solution containing 0.02% NaN_3_ at a flow rate 0.5 mL min^−1^ on the Sephadex G-100 column (internal diameter (i.d.) × *l* = 2.5 × 100 cm). The fraction collector (ISCO Retriever 500, Isco., Lincoln, NE) was used to collect the eluents, 6 mL per tube. The collection was stopped at the 100th tube. The calibration curve was established using the authentic dextrans having molecular masses 8.8, 40, 500, 2000 and 5000–40 000 kDa (Sigma, St Louis, MO, USA), respectively. The molecular mass distribution and the average molecular mass was calculated against this linear correlation between the logarithm of each standard molecular mass and the ratio the eluted to the void volume [36].

### 2.9. Analysis for Ratio Carbohydrate to Peptide Moiety

Different SP (D1–D6) was subjected to the Sephadex G-100 column separation. The Dubois' phenol-sulfuric acid colorimetric method [30] was followed to determine the carbohydrate content. Briefly, to each milliliter of the polysaccharide fraction 1 mL of phenol reagent (5%) and 5 mL of sulfuric acid were added. On cooling to the ambient temperature, the optical density was read at 490 nm. Alternatively, Coconnier et al. [37] was followed for the determination of peptido-moiety. The absorbance at 280 nm was taken straightforwardly for the six SP fractions (D1–D6) obtained from GPC.

### 2.10. Acid Hydrolysis

Three grams of each sample SP were accurately weighed and transferred separately into a 2 mL reaction vessel, to which 2 mL of 6 M HCl was added. The dissolved oxygen was purged off by nitrogen blowing for 10 min. The vessel was sealed, placed in the derivatization reactor, and heated at 110°C for 24 h until the peptide moiety was completely hydrolyzed. The hydrolyzed product was lyophilized. The desiccated product was re-dissolved in 0.3 mL of 0.01 M HCl to obtain the sample amino-acid mixture (AM).

### 2.11. Derivatization of Amino Acid and Extraction

An amount of 0.3 mL of authentic and AM (0.6 mL) solutions were respectively placed into a 3-mL reaction vessels, to which the internal standard solution of norleucine (0.01 mL, 10 mg mL^−1^) was added. When vigorously agitated, 0.1 mL of ethyl chloroformate and 1 mL of alcohol-pyridine were added and mixed thoroughly. To the mixture 2 mL of chloroform were added. When mixed well for 1 min to facilitate the derivatization and the extraction, water (0.7 mL) was added, shaken well, and left to stand for 5 min to facilitate phase separation. The supernatant was discarded. The lower layer (i.e., the chloroform layer) was transferred into a new tube and dehydrated with 0.1 g of anhydrous sodium sulfate. The dehydrated chloroform extract was transferred into the sample vessel and analyzed by GC/MS.

### 2.12. GC/MS Analysis

The GC/MS chromatography (Angilent 6890, Wilmington, DE, USA) installed with an FID detector and a column HP-5MS was used for GC/MS analysis. The HP-5MS column had a length *l* = 30 m with i.d. = 0.25 mm and film thickness = 0.25 *μ*m. The flow rate of carrier nitrogen gas was operated at 0.8 mL min^−1^. The temperature of the detector FID and the injection port was held at 305 and 300°C, respectively. The elution temperature was programmed initially at 50°C for 1 min, then raised at an elevation rate 10°C min^−1^ to 300°C and held at which for 6.5 min.

### 2.13. Assay for Antioxidative Capability—Preparation of SP Solutions

To 250 mg of SP (D1–D6) samples, 1 mL of 10% NaOH and sufficient double distilled water were added to make a final volume of 5 mL. The mixture was thoroughly agitated to facilitate the dissolution and centrifuged at 2500 × g for 10 min to remove the insoluble precipitate. The supernatant was collected (SP stock solution) for the following assays.

#### 2.13.1. Scavenging Capability for DPPH Radicals

Method of Blois [38], later modified by Shimada et al. [39], was followed for assay of the DPPH radicals. Briefly, to 1 mL of DPPH (methanolic DMSO solution, 0.5 mM, Sigma-Aldrich Co., MO, USA) an appropriate amount of each SP stock solution (20–100 *μ*L) or the standard was added. The mixture was thoroughly agitated and left to stand for 30 min. The absorbance was read at 517 nm using the BioMate 5 spectrophotometer (Thermo Electron Corporation, San Jose, CA, USA). DHA was used as the reference standard. The percent DPPH radical scavenging activity was calculated as directed [38, 39].

#### 2.13.2. Scavenging Capability for Hydroxyl- and Superoxide Anion Radicals

The modified method of Adom and Liu [40] modified by Nakajima [41] was used for the assay of antioxidant-capacity against the intracellular reactive oxygen species (ROS, including H_2_O_2_, •O_2_^−^ and •HO). Briefly, cells were seeded at a density of 2 × 10^3^ cells per well onto a 96-well plate and incubated at 37°C for 24 h in the humidified atmosphere containing 5% CO_2_. Each time the cell-culture medium was replaced fresh before any treatment with the soluble polysaccharide fraction of *D. indusiata* or the vehicle (DMEM containing 1% FBS) alone. On treatment for 1 h, 10 *μ*M of 5-(and-6)-chloromethyl-2′,7′-dichlorodihydrofluorescein diacetate (CM-H_2_DCFDA) (Molecular Probes, Eugene, OR, USA) was added to serve the radical probe. The incubation was further continued for 20 min at 37°C and the medium was replaced fresh to remove excess probe. In principle, CM-H_2_DCFDA (nonreactive to ROS) will be converted to dichlorodihydrofluorescein (DCFH) (reactive to ROS) by the intracellular enzyme esterase during incubation. The H_2_O_2_ or •O_2_^−^ will then oxidize the intracellular non-fluorescent DCFH to produce fluorescence active dichlorofluorescein (DCF). To generate H_2_O_2_ or •O_2_^−^, 1 mM H_2_O_2_ (Wako Pure, Osaka, Japan) or 1 mM KO_2_ (Aldrich Chemical Co., Milwaukee, WI, USA) was added. While ROS was still continuously persisting, the fluorescence was measured at 485/535 nm as the excitation/emission wavelengths using the Skan It RE (Varioskan Flash 2.4, Thermo Fisher Scientific, Waltham, MA, USA). Alternatively, for detection of the hydroxyl free radical •HO, 2-[6-(4′-amino) phenoxy-3H-xanthen-3-on-9-yl] benzoic acid (APF) (Daiichi Pure Chemicals, Tokyo, Japan) was used. Briefly, cells were incubated for 20 min at 37°C in Hanks/Hepes buffer solution containing APF (10 *μ*M). H_2_O_2_ was added to initiate the Fenton reaction. Finally iron (II) perchlorate hexahydrate (Wako Pure Chemicals, Osaka, Japan) was added. The fluorescence was measured at the excitation/emission wavelength 490/515 nm. The total intensity was calculated by integrating the scanned area under the DCFH.

### 2.14. Statistical Analysis

Data obtained in the same group were analyzed by Student's *t-*test with computer statistical software SPSS 10.0 (SPSS, Chicago, IL). Statistical analysis system software was used to analyze the variances. Duncan's multiple range tests were used to test their significances of difference between paired means. Significance of difference was judged by a confidence level of *P* < .05.

## 3. Results

### 3.1. Proximate Composition

The fruiting bodies of *D. indusiata* contained huge amount of carbohydrates and crude fibers reaching 46.89 and 28.65%, respectively. In contrast, it had only 6.07% of crude protein (Table 1). The SP yielded 37.5%, accounting for ∼80% (37.50/46.89 ≈ 0.8) of the total polysaccharides. The order of percentage yield (%w/w) was D4 (11.8) > D2 (9.3) > D1 (7.4) > D6 (6.6) > D3 (2.3) (calculated from Tables 1 and 2). Fraction D2 had the smallest protein to carbohydrate ratio (0.011), the order in increasing trend was: D4 (0.098) < D6 (0.203) < D1 (0.335) < D5 (2.011) < D3 (2.670). The mean MW ranged within 801–4656 kDa (Table 2). Among which fraction D3 had the smallest MW 801 kDa, D6 (MW 1375 kDa) the next, while D1 was the largest (4656 kDa) (Table 2).

### 3.2. Monosaccharide Composition

Ten kinds of monosaccharides were found in the SP of *D. indusiata*. Obeying the conventional naming rule, fraction D1 was designated glucogalactan; D3, the riboglucan, and both D5 and D6, the mannogalactans. Interestingly, huge amount of myo-inositol was simultaneously found in fractions D4, D2), and D1 (Table 3).

### 3.3. Antioxidative Capability

**Figure 4:**
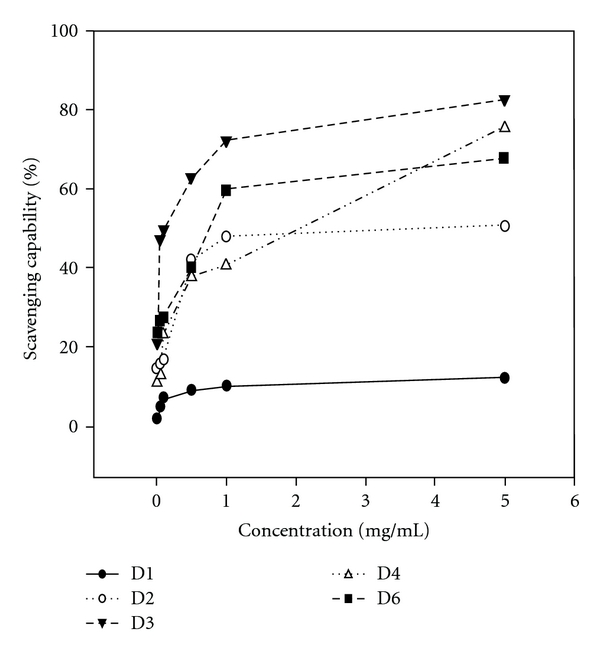
Scavenging capability of different polysaccharides fractionated from the fruiting bodies of *D. indusiata* for DPPH radicals. The dose ranged within 0–5.0 mg mL^−1^1 (upper). Values are expressed in means ± SD (*n* = 3).

**Figure 5:**
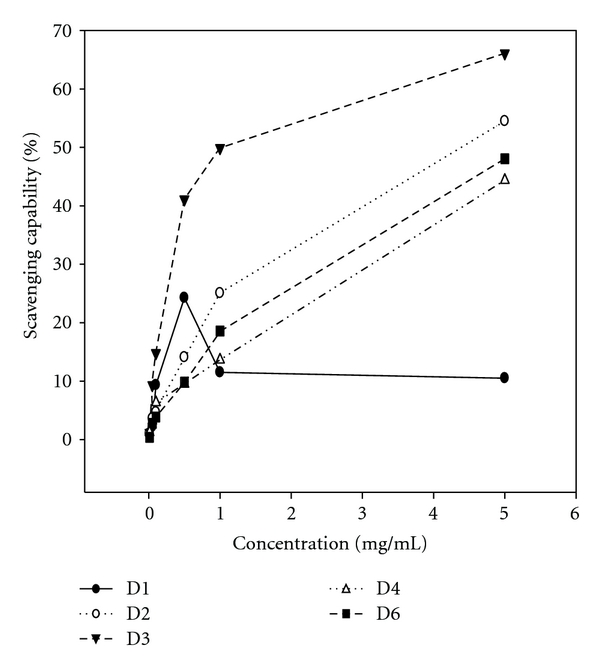
Scavenging capability of different polysaccharides fractionated from the fruiting bodies of *D. indusiata* for hydroxyl radicals. The dose ranged within 0–5.0 mg mL^−1^ (upper). Values are expressed in means ± SD (*n* = 3). Values are expressed in means ± SD (*n* = 3).

**Figure 6:**
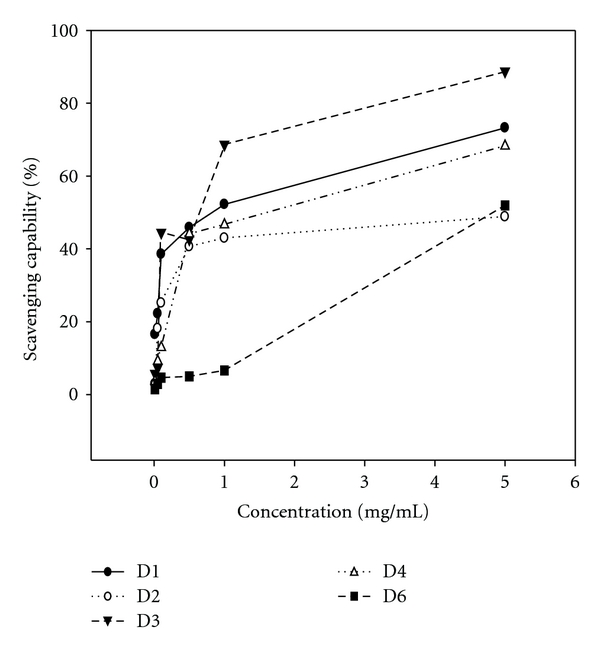
Scavenging capability of different polysaccharides fractionated from the fruiting bodies of *D. indusiata* for superoxide anion radicals. The dose ranged within 0–5.0 mg mL^−1^ (upper). Values are expressed in means ± SD (*n* = 3).

Fraction D3 at 1 mg mL^−1^ exhibited the most potent DPPH radical scavenging capability, D6 and D2 were the next, reaching 72, 60 and 47%, respectively. The order in strength was D3 > D6 > D2 > D4 > D1 (Figure 4). Regarding the scavenge for the hydroxyl radicals, fraction D3 also showed the most prominent bioactivity (reaching 50%) with the order D3 > D2 > D6 > D4 > D1 (Figure 5). Conversely, in scavenging superoxide anion radicals, the order changed to D4 (46%) ≈ D1 (44%) ≈ D2 (43%) > D3 (28%) > D6 (3%) (Figure 6). To further measure the crucial sensitivity, values of IC_50_ were compared. As seen, values of IC_50_ ranged within 0.11–3.93 mg mL^−1^ for DPPH, among which D3 was the most potent, and in contrast D1 almost had no effect toward DPPH and •OH free radicals (Table 4). To summarize, fraction D3 possessing the smallest MW (801 kDa) exhibited the most effective overall antioxidative potency.

## 4. Discussion

The fruiting bodies of *D. indusiata* contained huge amount of carbohydrates (46.89%). In parallel, its crude protein content was only 6.07%, implicating the possible peptidoglycan structure underling the main medicinal effect. Data revealed that only fractions D1, D2, D4 and D6 were peptidoglycan in nature. As a contrast, fractions D3 and D5 were typically characteristic of glycoproteins. Moreover, the high total SP content (37.44%) may also implicate an alternate therapeutic use. By insight of monosaccharide profile, fraction D1 was designated a glucogalactan; D3, a riboglucan; D5 and D6, mannogalactans. Fraction D4, having myo-inositol content 92.5%, almost was approaching the pure myo-inositol. Lin purified a polysaccharide xylose-containing Di-S2P from *D. indusiata* fruiting bodies. Di-S2P had MW 870 kDa [19], however xylose was undetected in D3. Worth noting, D4 and D6 uniquely contained allose (Table 3).

As well known, inositol epimerase governs the conversion of myo-inositol to chiro-inositol [42–44], an insulin dependent process related with immunological activity. Myo-inositol is an essential nutrient required for keratinocytes growth [45, 46]. The actual role of such an unusually high content of myo-inositol in *D. indusiata* acting as immunomodulator remains to further investigation. D3 possessed the most potent DPPH radical scavenging capability prevailing over the mannogalactan (D6), implicating the complicate phytochemical-therapeutics relationship [47]. As well cited, the binding of glucans having (1→3)-*β*-d-glucosan main frame with *β*-d-(1→6) branches onto the cell surface of cytotoxic macrophages, helper T and NK cells will trigger immunopotentiation [47, 48]. The degree of branching (DB) between 0.20 and 0.33 and the triple helical structures are important for immunopotentiating [47]. Conversely, some insoluble aggregates also had been reported to be more stimulatory than the soluble polymers [47]. A water-insoluble, alkali-soluble extracellular glucan (CO-1) (average MW *∼*632 kDa) isolated from *Cordyceps ophioglossoides* was shown to inhibit the Sarcoma 180 tumor [48].

To conclude, the fruiting bodies of *D. indusiata* contain huge amount of SP (37.5%) that exhibits MW ranging within 801–4656 kDa. Among which, the fraction D3 (the isoelectrically precipitated riboglucan from 2% NaOH) has the smallest MW 801 and is the most potent SP regarding the antioxidative capability. Furthermore, the huge amount of myo-inositol present can be relevantly associated with its additional immunobioactivity. Thus, we have confirmed our hypothesis that the bioactivity of *D. indusiata* is related in majority, if not entirely, to its soluble polysaccharides.

## Figures and Tables

**Table 1:** Proximate composition of the fruiting bodies of *D. indusiata* (Vent. Ex Pers.) Fish Phallaceae^a^.

Composition	Content (% w/w)
Moisture	10.55 ± 0.02
Crude ash	6.58 ± 0.01
Crude fat	1.26 ± 0.02
Crude fiber	28.65 ± 0.05
Crude protein	6.07 ± 0.03
Carbohydrate	46.89 ± 0.04

^a^Each value is expressed as mean ± SD (*n* = 3).

**Table 2:** Percent yield, mean molecular weight, and the content of carbohydrate and protein of the soluble polysaccharides obtained from the fruiting bodies of *D. indusiata* ^a^.

Extract	Yield (% w/w)	Carbohydrate (% w/w)	Protein (% w/w)	Mean MW (kDa)^b^
D1	7.4 ± 0.8	58.3 ± 3.5	19.5 ± 2.1	4656 ± 427
D2	9.3 ± 1.0	98.8 ± 5.6	1.1 ± 0.1	2118 ± 216
D3	2.3 ± 0.1	27.2 ± 1.5	72.6 ± 4.2	801 ± 82
D4	11.8 ± 1.1	90.7 ± 4.7	8.9 ± 1.5	2919 ± 258
D5	0.1 ± 0.0	27.4 ± 1.6	55.1 ± 2.8	Unanalyzed^c^
D6	6.6 ± 0.4	42.3 ± 2.4	8.6 ± 1.4	1375 ± 163

Total	37.5	—	—	—

^a^Weight-based percentage of soluble polysaccharides in 100 g of lyophilized fruiting body powder of *D. indusiata*. D1: the 3-fold ethanol precipitate from hot water extracts. D2: the 3-fold ethanol precipitate from 0.04 N HCl extracts. D3: the isoelectric precipitate from 2% NaOH extracts. D4: the 3-fold ethanol precipitate from 2% NaOH extracts. D5: the isoelectric precipitate from 10% KOH extracts; and D6: the 3-fold ethanol precipitate from 10% KOH extracts. Carbohydrate content (% w/w) was measured by the phenol-H_2_SO_4_ method. Protein content (% w/w) was determined by Bradford protein assay.

^b^Mean molecular weight.

^c^Unanalyzed due to insufficient quantity of sample.

**Table 3:** Pattern of soluble polysaccharides isolated from the fruiting bodies of *D. indusiata* (Vent. Ex Pers.) Fish Phallaceae^a^.

Sugar (mole%)	D1	D2	D3	D4	D5	D6
Rhamnose	1.0 ± 0.1^d^	0.2 ± 0.0^d^	13.6 ± 0.1^a^	0.3 ± 0.0^d^	1.8 ± 0.1^b^	1.1 ± 0.0^c^
Fucose	1.3 ± 0.1^c^	0.3 ± 0.0^d^	3.7 ± 0.0^a^	0.3 ± 0.0^d^	2.0 ± 0.1^b^	2.3 ± 0.0^b^
Ribose	1.4 ± 0.1^b^	0.1 ± 0.0^d^	24.3 ± 0.1^a^	0.4 ± 0.0^c^	ud	ud
Arabinose	1.6 ± 0.2^b^	0.7 ± 0.1^c^	3.3 ± 0.1^a^	0.8 ± 0.1^c^	ud	1.8 ± 0.0^b^
Xylose	ud	1.4 ± 0.1	ud	ud	ud	ud
Allose	ud	ud	ud	0.3 ± 0.0^b^	ud	2.2 ± 0.0^a^
Talose	ud	ud	ud	ud	ud	ud
Mannose	5.9 ± 0.3^b^	0.4 ± 0.1^d^	6.9 ± 0.1^b^	2.4 ± 0.2^e^	24.7 ± 0.3^a^	28.0 ± 0.2^a^
Galactose	14.2 ± 0.3^c^	19.7 ± 0.3^b^	2.9 ± 0.0^d^	2.0 ± 0.1^e^	66.2 ± 0.5^a^	62.0 ± 0.3^a^
Glucose	6.9 ± 0.3^b^	2.0 ± 0.1^d^	35.9 ± 0.2^a^	1.1 ± 0.0^e^	ud	2.7 ± 0.1^c^
Myo-inositol	67.9 ± 0.3^c^	76.5 ± 0.3^b^	9.4 ± 0.1^d^	92.5 ± 0.4^a^	5.3 ± 0.2^e^	ud

ud: undetected. Different superscripts in each column denote significant difference (*P* < .05) between fractions D1–D6.

^a^Weight-based percentage of polysaccharides extracted from 100 g of lyophilized fruiting body powder of *D. indusiata*. D1: the 3-fold ethanol precipitate from hot water extracts. D2: the 3-fold ethanol precipitate from 0.04 N HCl extracts. D3: the isoelectric precipitate from 2% NaOH extracts. D4: the 3-fold ethanol precipitate from 2% NaOH extracts. D5: the isoelectric precipitate from 10% KOH extracts; and D6: the 3-fold ethanol precipitate from 10% KOH extracts. Carbohydrate content (% w/w) was measured by the phenol-H_2_SO_4_ method. Protein content (% w/w) was determined by Bradford protein assay.

**Table 4:** The IC_50_ values of different soluble polysaccharide fractions obtained from the fruiting bodies of *D. indusiata* for scavenging the DPPH-, hydroxyl- and superoxide anion radicals^a^.

Polysaccharide fraction^a^	IC_50, DPPH_ (mg mL^−1^)	IC_50, •OH_ (mg mL^−1^)	IC_50, superoxide_ (mg mL^−1^)
D1	ud	ud	1.76
D2	3.93	4.39	ud
D3	0.11	1.02	0.64
D4	2.06	8.99	1.63
D6	0.75	5.63	4.79

ud: undetected.

^a^Weight-based percentage of polysaccharides extracted from 100 g of lyophilized fruiting body powder of *D. indusiata*. D1: the 3-fold ethanol precipitate from hot water extracts. D2: the 3-fold ethanol precipitate from 0.04 N HCl extracts. D3: the isoelectric precipitate from 2% NaOH extracts. D4: the 3-fold ethanol precipitate from 2% NaOH extracts. D5: the isoelectric precipitate from 10% KOH extracts; and D6: the 3-fold ethanol precipitate from 10% KOH extracts. Carbohydrate content (% w/w) was measured by the phenol-H_2_SO_4_ method. Protein content (% w/w) was determined by Bradford protein assay.
